# Kinetic analysis of cardiac dynamic ^18^F-Florbetapir PET in healthy volunteers and amyloidosis patients: A pilot study

**DOI:** 10.1016/j.heliyon.2024.e26021

**Published:** 2024-02-10

**Authors:** Haiyan Wang, Bolun Li, Zhe Wang, Xing Chen, Zhiwen You, Yee Ling Ng, Qi Ge, Jianmin Yuan, Yun Zhou, Jun Zhao

**Affiliations:** a Department of Nuclear Medicine, Shanghai East Hospital, School of Medicine, Tongji University, No. 150, Jimo Road, Shanghai, 200120, China; b Central Research Institute, United Imaging Healthcare Group Co., Ltd, Shanghai, 201807, China

**Keywords:** ^18^F-florbetapir, Cardiac amyloidosis, pSRTM, Pseudo-reference tissue, Quantitative analysis

## Abstract

**Objectives:**

This study aimed to explore the potential of full dynamic PET kinetic analysis in assessing amyloid binding and perfusion in the cardiac region using ^18^F-Florbetapir PET, establishing a quantitative approach in the clinical assessment of cardiac amyloidosis disease.

**Materials & methods:**

The distribution volume ratios (DVRs) and the relative transport rate constant (R_1_), were estimated by a pseudo-simplified reference tissue model (pSRTM2) and pseudo-Logan plot (pLogan plot) with kidney reference for the region of interest-based and voxel-wise-based analyses. The parametric images generated using the pSRTM2 and linear regression with spatially constrained (LRSC) algorithm were then evaluated. Semi-quantitative analyses include standardized uptake value ratios at the early phase (SUVR_EP_, 0.5–5 min) and late phase (SUVR_LP_, 50–60 min) were also calculated.

**Results:**

Ten participants [7 healthy controls (HC) and 3 cardiac amyloidosis (CA) subjects] underwent a 60-min dynamic ^18^F-Florbetapir PET scan. The DVRs estimated from pSRTM2 and Logan plot were significantly increased (HC *vs* CA; DVR_pSRTM2_: 0.95 ± 0.11 *vs* 2.77 ± 0.42, *t*’(2.13) = 7.39, P = 0.015; DVR_Logan_: 0.80 ± 0.12 *vs* 2.90 ± 0.55, *t*’(2.08) = 6.56, *P* = 0.020), and R_1_ were remarkably decreased in CA groups, as compared to HCs (HC *vs* CA; 1.08 ± 0.37 *vs* 0.56 ± 0.10, *t*’(7.63) = 3.38, *P* = 0.010). The SUVR_EP_ and SUVR_LP_ were highly correlated to R_1_ (r = 0.97, *P* < 0.001) and DVR(r = 0.99, *P* < 0.001), respectively. The DVRs in the total myocardium region increased slightly as the size of FWHM increased and became stable at a Gaussian filter ≥6 mm. The secular equilibrium of SUVR was reached at around 50-min p.i. time.

**Conclusion:**

The DVR and R_1_ estimated from cardiac dynamic ^18^F-Florbetapir PET using pSRTM with kidney pseudo-reference tissue are suggested to quantify cardiac amyloid deposition and relative perfusion, respectively, in amyloidosis patients and healthy controls. We recommend a dual-phase scan: 0.5–5 min and 50–60 min p.i. as the appropriate time window for clinically assessing cardiac amyloidosis and perfusion measurements using ^18^F-Florbetapir PET.

## 1 Introduction

Amyloidosis is a systemic disorder characterized by the extracellular deposition of misfolded and aggregated amyloid proteins into the beta-pleated-sheet structure [1]. Progressive deposition of amyloid fibrils could result in damage and dysfunction of multiple organ systems such as the heart, liver and lung [[2], [3], [4]]. Congestive heart failure caused by cardiac amyloidosis (CA) is the main cause of mortality and morbidity in CA [5]. The ‘gold standard’ for the confirmation of CA often involves the histopathological confirmation of myocardial biopsy, which is highly invasive. To date, multiple radiotracers have been shown to specifically bind to the amyloid plaques and visualize their changes in real-time. Promising results have also been demonstrated in detecting cardiac amyloid deposition by semi-quantitative ^18^F-Florbetapir PET/CT [[6], [7], [8]]. Despite various excellent targeted imaging techniques in conjunction with different radiotracers that have been implemented, CA remains underdiagnosed at present. Therefore, it is inevitable to discover and advance the current diagnostic strategies and provide better prognosis information.

Semi-quantitative measurement using standardized uptake value (SUV) was predominantly used in estimating the uptake of the radiotracer in the body. Previous CA studies by ^18^F-Florbetapir PET have demonstrated SUV method is a promising technique in the assessment of cardiac amyloid [[6], [7], [8]]. It is noteworthy that SUVs reflect the total activity that comprises not only the specific bio-pathophysiological information of interest but also free and non-specific signals. This uncertainty may result in bias and inaccuracy in quantitative measurements of the physiological parameters that reflect the tracer's characteristics. The absolute quantification by parameters derived from tracer pharmacokinetic modeling is a more robust approach compared to SUV for quantifying tracer uptake and utilization [9]. Tracer kinetic modeling for instance the simplified reference tissue model (SRTM), has been predominantly adopted in radioligand-receptor PET for the quantification of tracer binding as it is non-invasive [10]. The prominent features of SRTM are attributed to its high robustness and computationally efficient in estimating kinetic parameters, as well as the ability to generate parametric images of ligand-receptors with the application of linear regression with spatial constraint (LRSC) algorithm [11]. Despite quantitative tracer kinetic analyses that have been extensively studied in the brain, especially in AD [[12], [13], [14], [15]], their application on CA is critically lacking. In this pilot study, we proposed the utilization of a pseudo-simplified reference tissue model (pSRTM) and a pseudo-Logan plot, employing the kidney as a pseudo-reference tissue to determine the discriminating ability between healthy volunteers and CA patients, by estimating DVR and R_1_ from a 60-min cardiac ^18^F-Florbetapir PET/MR scan. Based on the quantitative dynamic PET analysis, we optimized data acquisition protocols for clinical cardiac ^18^F-Florbetapir PET.

## 2 Materials and methods

### 2.1 Participants

The study population consisted of 7 healthy controls (HC) (age 65.00 ± 10.52) and 3 CA subjects (age 66.67 ± 8.96). All participants underwent a 60-min cardiac dynamic PET scan. The CA group's inclusion criteria encompass individuals who are clinically suspected of having cardiac amyloidosis. The HC group's inclusion criteria are individuals with no history of heart disease, normal results in electrocardiogram (ECG) and cardiac function tests. Conversely, exclusion criteria are established based on contraindications for MR Examination, including situations following pacemaker and heart valve replacement. Furthermore, individuals with limitations related to body position or those with a weakened physical state, unable to tolerate the examination, are excluded. Additionally, individuals exhibiting allergies to contrast media are not considered eligible for participation in the study. The diagnostic criteria for CA include a comprehensive evaluation using various modalities. Firstly, the medical history, ECG, echocardiography, cardiac magnetic resonance (CMR), and cardiac biomarker examinations are utilized to suggest the presence of CA. Secondly, confirmation through histological biopsy is essential, with amyloid deposition being specifically identified through positive Congo red staining in extracardiac tissues, such as abdominal wall fat and lip glands. Additionally, the detection of plasma monoclonal immunoglobulin in urine, along with serum free light chain (kappa and lambda) quantitative assessments or ratio abnormalities (kappa/lambda <0.26 or >1.65), constitutes crucial diagnostic parameters for CA. The type of amyloidosis (light-chain amyloidosis (AL) or transthyretin amyloidosis (ATTR)) was not classified. The baseline characteristics of CA patients were included in Supplemental Table 1. All subjects were provided with written informed consent to undergo procedures approved by Shanghai East Hospital, Tongji University (Shanghai 20012, China).

### 2.2 Image acquisition

Simultaneous ^18^F-Florbetapir PET/MR scans were performed on an integrated PET/MR system (uPMR 790, United-Imaging Healthcare, Shanghai) equipped with 12-channel phase-array body surface coil. A segmented MR-based attenuation correction map for ungated PET was generated by using the same respiration-gated Dixon sequence [16]. PET was started immediately after an intravenous bolus injection of ^18^F-Florbetapir of 9.04 ± 1.89 mCi (mean ± SD, hereafter).

### 2.3 Image reconstruction

PET was simultaneously acquired with all MRI sequences and saved in list mode format for reconstruction. The PET list mode data were first performed with 511 KeV energy drift correction for the temperature-induced counting loss during an extensive MRI scan. Dynamic PET images (matrix size 192 × 192, field of view (FOV) = 35 cm, voxel size = 1.82 mm × 1.82 mm × 2.78 mm) were reconstructed in 12 frames of 10s, 6 frames of 20s, 6 frames of 60s, 5 frames of 120s, 8 frames of 300s, 12 frames of 10s, with a total of 37 frames using the ordered subset-expectation maximization (OSEM) algorithm (three iterations and 20 subsets with time-of-flight (TOF) and point-spread function (PSF) including corrections for random coincidences, dead-time, scatter and attenuation.

### 2.4 PET image processing

The uptake of ^18^F-Florbetapir in the left ventricle (LV) myocardium was visually performed using static PET images from 0 to 60 min post-injection (p.i.). A total of 18 regions of interest (ROIs) including 17 segments of the LV were manually oriented and adjusted, and then automatically segmented by using the PCARD model in PMOD (version 4.2, PMOD Technologies LLC., Zurich, Switzerland). The ROIs delineation of the left and right kidney were manually drawn based on the first 5-min mean images. Time activity curves (TACs) were obtained by applying ROIs to 0 and 60 min dynamic images for kinetic analysis. The ROIs were also applied directly to parametric images for the estimation of DVR and R_1_.

### 2.5 Kinetic modeling and semi-quantitative analysis

#### 2.5.1 pSRTM2

The reference tissue model is derived from the classical compartment model theory by using reference tissue TAC instead of plasma input [10]. SRTM supposes that the tracer concentration in reference and target can be modeled by a single compartment [17]. SRTM consists of 3 parameters: transport rate ratio from target to reference tissue (R_1_), efflux rate constant from free plus nonspecific compartment to blood (k_2_, 1/min), and distribution volume ratio between the target regions and reference tissue (DVR) [18]. According to the assumption of SRTM, pSRTM2 uses fewer model parameters that provide reduced noise in the parameters or parametric images estimated from high noise levels of tissue kinetics [19]. The pSRTM2 includes R_1_, k_2R,_ and DVR parameters, where k_2R_ (1/min) is the efflux rate from reference tissue to blood, and the parameters of pSRTM2 can be estimated by simultaneous model fitting to the TACs for all ROIs [14,17]. In this study, the parameters of the pSRTM2 were estimated by simultaneously fitting to the TACs for 17 segments of cardiac and total myocardium.

(1) $$\sum_{j=1}^{N}\sum_{i=1}^{M}w_{i}{\left(C^{j}\left(t_{i}\right)-f_{pSRTM2}\left(t_{i}|R_{1}^{j},k_{2R},{DVR}^{j}\right)\right)}^{2}$$

The pSRTM2 is given by the operational Eq. (1). Where *M* is the number of frames for the dynamic PET scan, *N* is the number of ROI TACs, $t_{i}$ is the mid time of i th frame, $w_{i}$ is the duration of i th frame, and $C^{j}\left(t_{i}\right)$ is the measured j th ROI's tracer concentration at i th frame. The j th ROI's tracer concentration at time $t_{i}$ is determined by $f_{pSRTM2}$ with the parameters $R_{1}^{j}$ and ${DVR}^{j}$ for j th ROI and the coupled parameter k_2R_.

The parametric images of R_1_ and DVR were generated by pSRTM2 using LRSC algorithm with a 3-dimensional Gaussian filter with full width at half maximum (FWHM) varying from 0 (regular weighted linear regression) to 12 mm to minimize the noise-induced underestimation in DVR [14].

#### 2.5.2 Logan plot

The Logan plot is reliable and widely used to estimate DVR for the low noise level of tissue kinetics. The standard Logan plot with reference tissue input is given by the operational Eq. (2) [20]:

(2) $$\frac{\int_{0}^{t}C_{T}\left(s\right)ds}{C_{T}\left(t\right)}=DVR\frac{\int_{0}^{t}C_{REF}\left(s\right)+\frac{C_{REF}\left(t\right)}{k_{2R}}}{C_{T}\left(t\right)}+\tau \;for\;t>t^{*}$$

Where the t* = 20 min, and k_2R_ was determined by the pSRTM2 model fitting.

### 2.6 Semi-quantitative analysis

Standardized uptake value ratio (SUVR) was calculated as the target to reference tissue tracer concentration ratio over a time frame after tracer injection. In this study, SUVR at the early phase (SUVR_EP_) was calculated from 0.5 to 5 min and the late phase (SUVR_LP_) was calculated from 50 to 60 min. The SUVR_EP_ and R_1_ represent relative cardiac blood flow or perfusion, while SUVR_LP_ and DVR reflect the tracer-specific binding to amyloid proteins [21].

### 2.7 Statistical analysis

Continuous variables with normal distribution were presented as mean ± SD and were compared between groups by the independent *t*-test or one-way analysis of variance (ANOVA) Pearson correlation analysis and simple linear regression were used to identify correlations. Statistical significance was defined as a *P* < 0.05 with two sides. Data were analyzed using SPSS software, version 24.0 (SPSS Inc. IBM, Armonk, NY, USA).

## 3 Results

### 3.1 ROI-based kinetic analysis using the kidney as pseudo-reference tissue

To evaluate the tracer kinetics in kidney, the statistics of k_2R_ of the kidney determined *via* ROI TAC-based simultaneous pSRTM2 fitting were analyzed. We observed that the tracer kinetics in the kidney are comparable between CA and HC groups. The k_2R_s for the kidney between the two groups were not statistically significant (HC *vs* CA; 0.18 ± 0.23 *vs* 0.24 ± 0.06, *t*’(7.50) = 0.68, *P* = 0.518).

**Fig. 1:**
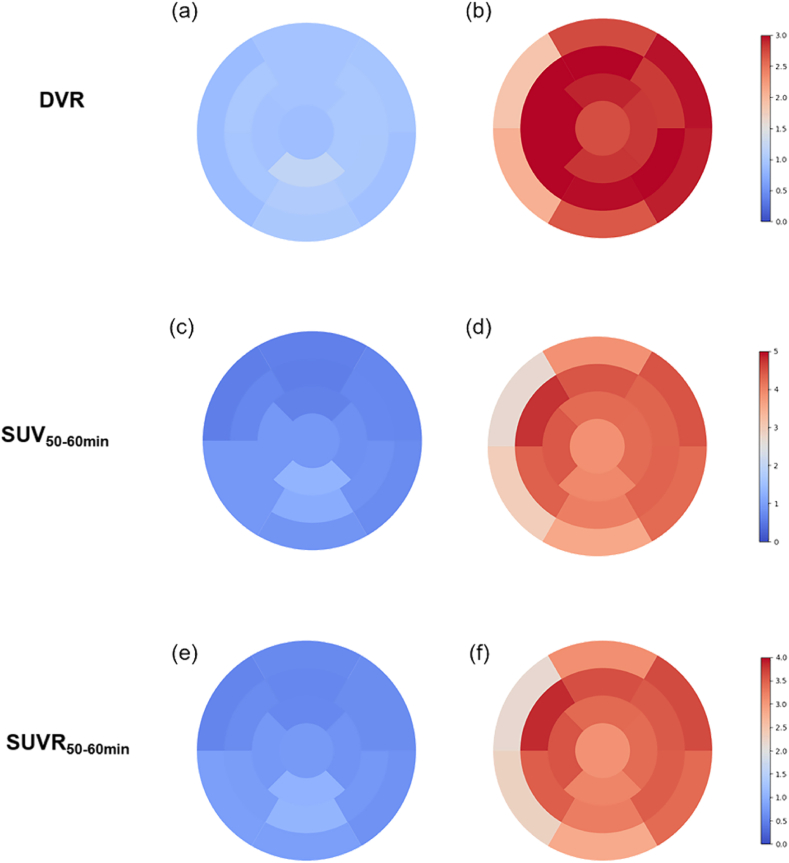
Bull's eye plot of DVR from pSRTM2, SUV_50–60min,_ and SUVR_50–60min_ in HC (a, c, e) and CA group (b, d, f) respectively.

For the assessment of amyloid distribution, the left ventricle was divided into 17 segments. The result of bull's eye plot showed HC demonstrated a significantly lower DVR (Fig. 1(a)), SUV_50–60min_ (Fig. 1(c)), and SUVR_50–60min_ (Fig. 1(e)) values than the CA group (Fig. 1(b), (d), 1(f)), respectively. The DVR, SUV_50–60min_, and SUVR_50–60min_ values for the total myocardium are 2.77 ± 0.42, 4.02 ± 1.25, and 3.23 ± 0.77 in the CA group and 0.95 ± 0.11, 0.82 ± 0.23, and 0.71 ± 0.15 in the HC group, respectively. Furthermore, we found that there was no large variability visually among the 17 segments in the CA group.

**Fig. 2:**
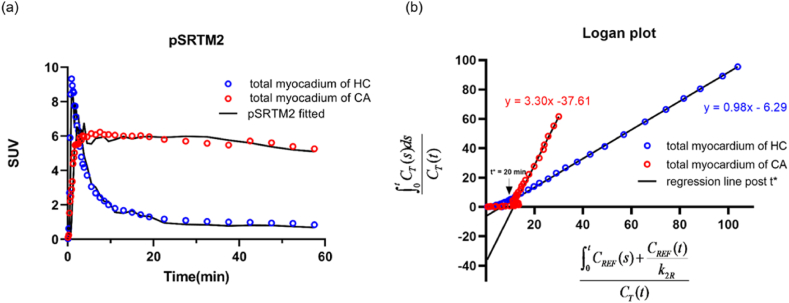
Kinetic modeling (pSRTM2(a), Logan plot(b)) using kidney reference tissue for representative CA patient and HC subject.

TACs and model fitting of total myocardium for CA (61-year-old female) and HC (77-year-old female) exemplar individuals were depicted in Fig. 2. For the HC and CA exemplar participants, the total myocardium SUV TAC peaked at 0.96 and 7.5 min respectively, and maintained a nearly constant level after 20 min p.i. The pSRTM2 fitting using the kidney as pseudo-reference tissue fits well with the measured total myocardium TAC (Fig. 2(a)). The parameters R_1_ and DVR were estimated for the total myocardium region. In Fig. 2(b), the linear segments of the corresponding Logan plot were started from t* = 20min. The pSRTM2 fitting is comparable to the Logan plot in terms of overall performance.

**Fig. 3:**
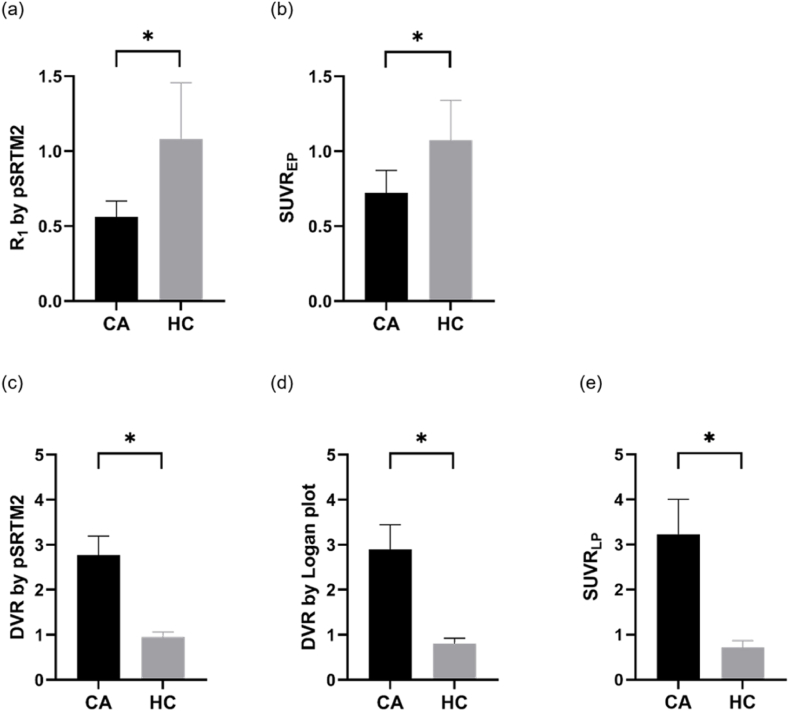
R_1_ generated by pSRTM2 (a) model fitting, SUVR_EP_ (b), DVR generated by pSRTM2 (c) and Logan plot (d) model fitting and SUVR_LP_ (e) of total myocardium region for CA and HC groups. Asterisks denote significant differences with HC group (*P < 0.05).

**Fig. 4:**
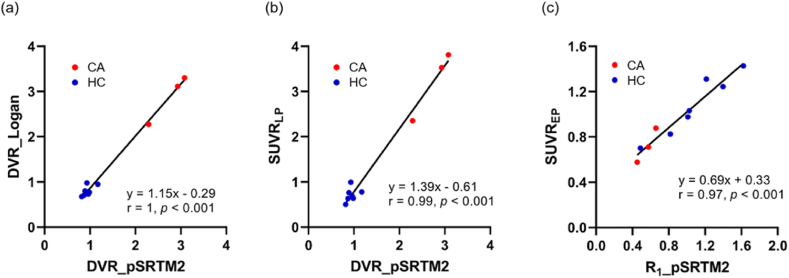
Positive linear correlation between DVR from pSRTM2 and DVR from Logan plot (a). Positive linear correlation between ROI estimates DVR from pSRTM2 and SUVR_LP_ (b). Positive linear correlation between ROI estimates R_1_ from pSRTM2 and SUVR_EP_ (c).

Both R_1_ from pSRTM2 (HC *vs* CA; 1.08 ± 0.37 *vs* 0.56 ± 0.10, *t*’(7.63) = 3.38, *P* = 0.010) and SUVR_EP_ (HC *vs* CA; 1.07 ± 0.27 *vs* 0.72 ± 0.15, *t*’(6.84) = 2.66, *P* = 0.033) in the whole myocardium region using the kidney as pseudo-reference tissue reveals substantial differences between the HC and CA groups (Fig. 3(a–b)). We found that the HC group had significantly lower DVRs (HC *vs* CA; DVR_pSRTM2_: 0.95 ± 0.11 *vs* 2.77 ± 0.42, *t*’(2.13) = 7.39, P = 0.015; DVR_Logan_: 0.80 ± 0.12 *vs* 2.90 ± 0.55, *t*’(2.08) = 6.56, *P* = 0.020) and SUVR_LP_ (HC vs CA; 0.71 ± 0.15 *vs* 3.23 ± 0.77, *t*’(2.07) = 5.59, *P* = 0.028) than the CA group (Fig. 3(c–e)). We considered the pSRTM2 as a better standard theoretically, the Logan plot, SUVR_EP_ and SUVR_LP_ showed a comparable ability to discriminate between CA and HC subjects. We also found significant positive linear correlations between DVRs from pSRTM2 and Logan plot (Fig. 4(a)), DVR from pSRTM2 and SUVR_LP_ (Fig. 4(b)), R_1_ from pSRTM2 and SUVR_EP_ (Fig. 4(c)).

### 3.2 Parametric imaging with LRSC

**Fig. 5:**
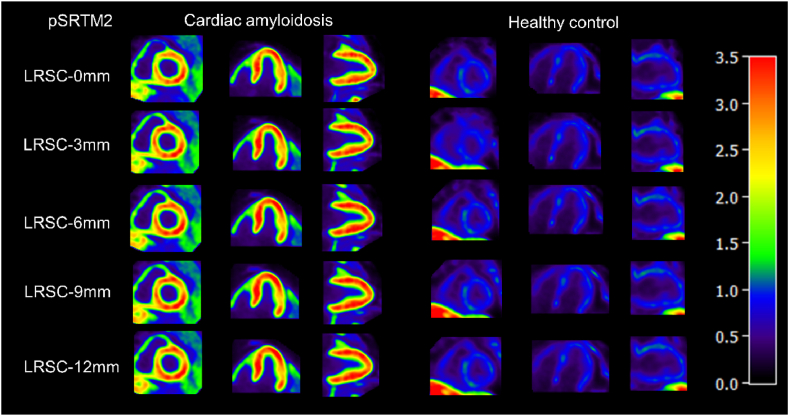
Representative DVR parametric images for cardiac amyloidosis subject and healthy control subject. Parametric images of DVR were generated from ^18^F-Florbetapir dynamic PET using the pSRTM2 with linear regression (LR), and linear regression with and without spatially constrained (LRSC) algorithm with 3-dimensional Gaussian filters with FWHM varying from 3 to 12 mm, where the spatial resolution of reconstructed PET is 2.9 mm FWHM.

The DVR parametric images were generated by the pSRTM2 using the LRSC algorithm as a function of the FWHM of the Gaussian filter (Fig. 5). The DVRs for the total myocardium region of parametric images from pSRTM2 with kidney as pseudo-reference tissue for the CA exemplar in 0 mm, 3 mm, 6 mm, 9 mm, 12 mm FWHM are 2.22, 2.29, 2.35, 2.37, and 2.37 respectively. For the HC exemplar, the DVRs for the total myocardium region in 0 mm, 3 mm, 6 mm, 9 mm, and 12 mm FWHM are 0.75, 0.76, 0.76, 0.76, and 0.76 respectively. We found a significant difference in myocardium region in pSRTM2 DVR parametric images between CA and HC groups, as well as comparable DVRs in different sizes of FWHM for HC participants were observed (Table 1). The DVRs in the total myocardium region increased slightly as the size of FWHM increased and became stable at a Gaussian filter ≥6 mm.

**Table 1:** The comparison of DVRs for the total myocardium region estimated from parametric images using pSRTM2 with different FWHM of 0 mm, 3 mm, 6 mm, 9 mm, and 12 mm.

	DVR_0 mm	DVR_3 mm	DVR_6 mm	DVR_9 mm	DVR_12 mm	Statistics
HC	0.51 ± 0.26	0.52 ± 0.25	0.53 ± 0.24	0.53 ± 0.24	0.53 ± 0.24	F(4, 30) = 0.014; P = 1.000
CA	2.03 ± 0.27	2.08 ± 0.30	2.12 ± 0.34	2.15 ± 0.36	2.16 ± 0.37	F(4, 10) = 0.088; P = 0.984
Statistics	t(8) = 8.48; P < 0.001	t(8) = 8.52; P < 0.001	t(8) = 8.57; P < 0.001	t(8) = 8.59; P < 0.001	t(8) = 8.60; P < 0.001	

**Fig. 6:**
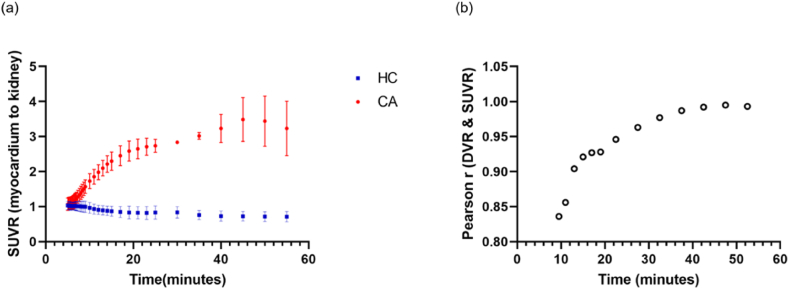
The change of mean SUVR (myocardium to the kidney) in the CA and HC group for every 10 min (a). The correlation of DVR and SUVR over time (b).

To further evaluate the optimal time window for ^18^F-Florbetapir PET-based CA clinical diagnosis, we calculated the Pearson correlation coefficients between DVR and a series of SUVR acquired at different time points with interval of 10 min (Fig. 6(b)). As well as the mean SUVR changes in CA and HC groups (Fig. 6(a)). A strong correlation was found between DVR and SUVR, especially between 50 and 60 min. In addition, the secular equilibrium of SUVR was reached at around 50 min p.i. time for both CA and HC groups (Fig. 6(a)). Moreover, the significant difference between CA and HC groups was also found using SUVR at notably 50–60 min imaging windows. Therefore, we suggest 50-min p.i. time would be an appropriate clinical diagnosis time window for ^18^F-Florbetapir PET imaging.

## 4 Discussion

**Fig. 7:**
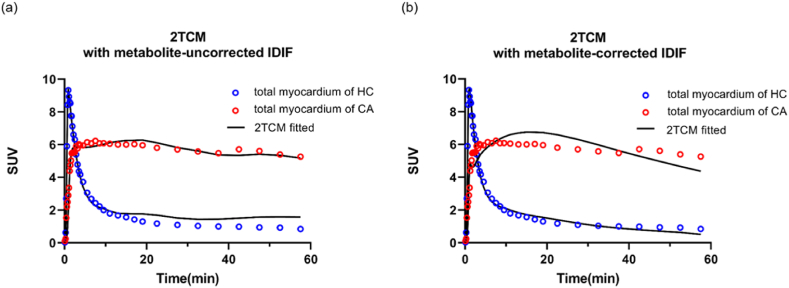
The Kinetic modeling (2-Tissue Compartment Model with metabolite-uncorrected image-derived input function (a), 2-Tissue Compartment Model with metabolite-corrected image-derived input function (b)) for representative CA patient and HC subject.

A major barrier to expanding studies that use ^18^F-Florbetapir imaging to assess the potential role of amyloidosis in the body is the rapid metabolization of ^18^F-Florbetapir in blood and other organs. An established approach to enhance the kinetic quantification of ^18^F-Florbetapir involves combining dynamic PET imaging with arterial blood sampling, considered the gold standard [13,22]. However, the invasive character of this method restricts its widespread clinical application [23]. To address this limitation, alternative methods such as the image-derived input function (IDIF) have been developed. Given the swift metabolism and kinetics of ^18^F-Florbetapir, especially at 10 min p.i., a population-based metabolite fraction function could be employed to correct for metabolites [13]. To validate this approach, we compared the population-based metabolite-corrected IDIF against direct IDIF using 2-tissue-compartment model (2TCM) fitting. Due to the affection of metabolites in blood, 2TCM using metabolite-uncorrected blood IDIF exhibited poor fit after 17 min, particularly in the HC exemplar (Fig. 7(a)). However, the poor fit persisted even after the population-based metabolite correction for blood input function (Fig. 7(b)). Our study revealed that using a population-based metabolite fraction function to correct the IDIF did not yield improvement. The fact that the same correction was applied to all subjects in the study suggests that there is inter-subject variability. The chosen population-based metabolite fraction function should be determined from the population under study in terms of age, sex, body weight and clinical condition [24]. Inter-subject variability is hardly ever insignificant even among groups of comparable subjects [25], and the presence of occasional outliers is to be anticipated. Therefore, we explored the use of pSRTM2 with a pseudo-reference tissue, eliminating the need for a blood input function to further quantify amyloid deposition in the cardiac region.

We have proposed that the kidney could be a choice to serve as a pseudo-reference tissue for ^18^F-Florbetapir quantification *via* pSRTM2 and Logan plot methods based on our study population. A pseudo-reference region would not differ between comparison groups; for example, one could compare the ratio of the target to the pseudo-reference region in patients versus controls [26]. In a study by Vomacka and colleagues, a pseudo-reference region was defined for their patient group based on the identification of the least disease-affected region, as compared to six HCs [27]. Identifying a pseudo-reference region in our study proved challenging due to the potential impact of amyloid deposition on multiple organ systems [[2], [3], [4]]. Although renal amyloidosis is well-described, however, to the best of our knowledge, the detection of amyloid deposition in the kidney among CA patients has not been previously reported. Furthermore, our study group did not reveal any instances of renal amyloid deposition, and tracer kinetics in the kidneys showed no significant differences between CA and HC groups. Therefore, we selected the kidney as the pseudo-reference tissue, given its absence of amyloid deposition for quantifying cardiac ^18^F-Florbetapir PET. However, if mild renal amyloid deposition is observed in individuals with CA, it could diminish the distinctions and contrasts in DVR between the CA and HC group, but not likely to alter statistical significance as high DVR contrast between two groups showed in the study. Continued research, as well as ongoing validation efforts, are imperative for the exploration and refinement of our approach in future studies.

Quantitative parameters R_1_ and DVR, as well as their corresponding semi-quantitative measures, SUVR_EP_ and SUVR_LP,_ could be used to discriminate CA from the HC group. In the CA group, we found significantly lower R_1_ while contradictorily higher DVR compared to the HC group, indicating that CA individuals with elevated amyloid deposition may experience a decreased myocardial perfusion rate compared to HC. It is noteworthy that reduced myocardial perfusion can be triggered by extracellular amyloid infiltration that interrupts intramyocardial vessels [28]. Since CA is a systemic disease, myocardial perfusion impairment may also persistently cause dysfunction of other vital organs, therefore the non-invasive quantitative parameters, R_1_ and DVR may be advantageous in the early detection of cardiac abnormalities and malfunction. In fact, R_1_ has been widely applied to quantitatively measure the relative cerebral perfusion of ^18^F-Flortaucipir [14], ^18^F-Florbetaben [29] and ^11^C-PiB [30] in many brain research. Although limited literature on the application of R_1_ on cardiac perfusion, our present study indicated that R_1_ derived from pSRTM2 could be used to quantify relative cardiac perfusion, which may serve as a functional perfusion parameter for clinical quantification.

Semi-quantitative measurements such as the ratiometric SUVR method have been extensively employed in previous ^18^F-Florbetapir studies to distinguish CA from HC. A long scan duration of 60 min has been performed in previous work by Dorbala and colleagues where multiple semi-quantitative variables including myocardial retention index, and target-to-background ratio were used to allow the discrimination of CA and HC [6]. Despite its simplicity and convenience in clinical practice, semi-quantitative measurements are vulnerable to various biological and technical factors that affect the quantification accuracy. Different from the previous study, our study performed kinetic modeling using pSRTM2 by deriving a quantitative parameter, DVR that allows the quantification of amyloid *in vivo*.

Furthermore, we also systematically evaluated both SUVR_EP_ and SUVR_LP_ to observe SUVR trends in both groups and their correlation with DVR at different time windows. Based on the trend of Pearson r, we noticed that DVR is highly correlated with SUVR at approximately 50–60 min. Since long dynamic PET acquisition may not be feasible in all clinical practice, hence we recommend SUVR_EP_ and SUVR_LP_ may be an appropriate semi-quantitative approach that is alternatively equivalent to quantitative parameters that reflects myocardial perfusion and amyloid distribution volume of CA.

It is important to consider the limitations of our study. The results obtained were derived from a small sample size, and the limited data was not adequate to reflect comprehensive conditions related to CA. This pilot study primarily focused on the evaluation of ^18^F-Florbetapir PET quantification and kinetic modeling performance *via* pSRTM2 and Logan plot in the clinical task of discriminating CA from HC. As illustrated in Fig. 3, our findings reveal a substantial difference in DVR between patients and healthy individuals. It's worth noting that the patients included in the study have a severe CA. Further validation, encompassing a larger sample size and including cases with varying degrees of severity, is crucial for confirming the effectiveness of our approach. It is also worthwhile to collect and validate different types of CA, such as AL and ATTR.

## 5 Conclusion

In conclusion, the utilization of pSRTM2 with the kidney as a pseudo-reference tissue showed its discriminative efficacy in detecting differences between the CA and HC groups by calculating DVR and R_1_. In addition, the calculations of SUVR_EP_ and SUVR_LP_ using the kidney as a pseudo-reference tissue provided comparable ability in detecting differences between the two groups. We recommend a dual-phase scan time window of 0.5–5 min and 50–60 min p.i. for the clinical diagnosis of CA using ^18^F-Florbetapir PET. Our pilot study advocates for an enhanced quantitative approach to precision imaging with ^18^F-Florbetapir in CA studies, emphasizing the need for further validation through the inclusion of a larger and more diverse sample, incorporating cases with different levels of severity.

## Funding

This study was funded by the Project of 10.13039/501100003399Science and Technology Commission of Shanghai Municipality (19DZ1930703).

## Ethical approval

The study involving human participants was in line with the principles of the ethics committee in East Hospital Affiliated to Tongji University (Shanghai 20012, China). [2020] number 085.

## Consent to participate

Informed consent was obtained from all individual participants included in the study.

## Availability of data

The data could be obtained from the corresponding author upon request.

## Consent to publish

Not applicable.

## Data availability statement

The data associated with our study have not been deposited in a publicly available repository. Data will be made available on request.

## CRediT authorship contribution statement

**Haiyan Wang:** Writing – review & editing, Writing – original draft, Visualization, Investigation, Formal analysis, Data curation, Conceptualization. **Bolun Li:** Writing – review & editing, Writing – original draft, Validation, Methodology, Investigation, Formal analysis, Conceptualization. **Zhe Wang:** Writing – review & editing, Investigation, Formal analysis, Data curation. **Xing Chen:** Writing – review & editing, Investigation, Formal analysis, Data curation. **Zhiwen You:** Writing – review & editing, Investigation, Formal analysis, Data curation. **Yee Ling Ng:** Writing – review & editing, Writing – original draft, Validation, Investigation. **Qi Ge:** Writing – review & editing, Validation, Methodology, Formal analysis. **Jianmin Yuan:** Writing – review & editing, Supervision, Conceptualization. **Yun Zhou:** Writing – review & editing, Validation, Supervision, Resources, Project administration, Methodology, Conceptualization. **Jun Zhao:** Writing – review & editing, Supervision, Resources, Project administration, Funding acquisition, Data curation, Conceptualization.

## Declaration of competing interest

The authors declare that they have no known competing financial interests or personal relationships that could have appeared to influence the work reported in this paper.
