# Supplementary material for: Kinetic analysis of cardiac dynamic 18F-Florbetapir PET in healthy volunteers and amyloidosis patients: A pilot study
Source: Heliyon. 2024 Feb 10;10(4):e26021. doi: 10.1016/j.heliyon.2024.e26021 (PMC10875429; doi:10.1016/j.heliyon.2024.e26021)
Supplement: Multimedia component 1 [file mmc1.pdf]

Supplemental data

Supplemental Table 1. Baseline characteristics of the CA patients.

Patient Number	Age	Symptoms	Electrocardiogram	Echocardiogram	Cardiac magnetic resonance	Positron emission tomography	Pathology/Laboratory examination	Survival time
Patient 1	61	a history of hypertension for 2 years, chest tightness and shortness of breath for 40 days after activity, and aggravated for 15 days	Sinus rhythm, possible recent anterior myocardial infarction, abnormal T-wave	Left and right atria were enlarged, ventricular septum and left ventricular wall thickened, left ventricular systolic function was normal (EF 60%) left ventricular diastolic function decreased	The shape of the heart is enlarged, the chambers of the two atria are enlarged, and the walls of the chambers of the two ventricles are thickened. Extensive delayed reinforcement of the left and right ventricular walls	^{18}F-Flobetapir distribution in the left ventricular wall, SUVmax = 7.91. The right ventricular wall with slightly distribution , SUVmax = 3.8	Labial gland and gastric mucosa biopsy were positive for Congo red staining	180 days

Patient 2	62	limb weakness, palpitation shortness of breath more than 8 months, gradually aggravated	Low voltage, poor R-wave increment	Left ventricular thickening, left ventricular systolic function normal (EF 60%) left ventricular diastolic function decreased	Multiple ventricular wall segments (left inferior middle segment + base segment, anterior wall + lateral parapical segment) were poorly perfused, and left and right ventricular walls were delayed strengthened	^{18}F-Flobetapir distribution in the left (SUVmax = 4.79)and right (SUVmax = 2.64)ventricular walls, and ^{18}F-Flobetapir distribution in both lungs	Urine k light chain 46.5 mg/L, urine λ light chain 2370 mg/L, blood k light chain 8.07 mg/L, blood λ light chain 2600 mg/L, k/λ = 0.003	45 days
Patient 3	77	Multiple myeloma for more than 4 years, multiple chemotherapy treatment, repeated lower limb edema for more than 1 month, aggravated with chest tightness and shortness of breath for 10 days	Sinus rhythm, S-T elevation, T-wave change	The left and right atria were enlarged, the basal segment of the ventricular septum was thickened, left ventricular systolic function was normal (EF 64%), and left ventricular diastolic function decreased	The left ventricular wall showed diffuse thickening, enlargement of the left and right atria, and enhancement of the left ventricular wall in the uneven delayed period, especially subendocardial enhancement	^{18}F-Flobetapir distribution in left ventricle , SUVmax = 5.79; The radioactivity distribution in the right ventricular wall with slightly distribution, SUVmax = 2.01	Urine K light chain 46.5 mg/L, urine λ light chain 3.66mg/L, blood K light chain 28.07 mg/L, blood λ light chain 0.98mg/L, k/λ=28.6	14 days
